# Effects of depression, dementia and delirium on activities of daily living in elderly patients after discharge

**DOI:** 10.1186/s12877-019-1294-9

**Published:** 2019-10-11

**Authors:** Ching-Fu Weng, Kun-Pei Lin, Feng-Ping Lu, Jen-Hau Chen, Chiung-Jung Wen, Jui-Hua Peng, Ailun Heather Tseng, Ding-Cheng Chan

**Affiliations:** 1 Department of Internal Medicine, Division of General Chest Medicine, Hsinchu Cathay General Hospital, Hsinchu, Taiwan; 2 Department of Geriatrics and Gerontology, National Taiwan University Hospital, Taipei, Taiwan; 3 Department of Internal Medicine, National Taiwan University Hospital, Taipei, Taiwan; 4 Department of Family Medicine, National Taiwan University Hospital, Taipei, Taiwan; 5 Systems Biology and Bioinformatics, National Central University, Taoyuan, Taiwan; 6 Superindendent Office, National Taiwan University Hospital Chu-Tung Branch, No.52, Zhishan Rd., Zhudong Township, Hsinchu County, Taiwan 310

**Keywords:** Functional status, Geriatric syndrome, Hospitalization

## Abstract

**Background:**

The three geriatric conditions, depression, dementia and delirium (3D’s), are common among hospitalized older patients and often lead to impairments of activities of daily living. The aim of this study is to explore the impact of depression, dementia and delirium on activities of daily living (ADLs) during and after hospitalization.

**Methods:**

A prospective cohort study was conducted between 2012 and 2013 in a tertiary medical center in Taiwan. Patients who aged over 65 years and admitted to the geriatric ward were invited to this study. Geriatric Depression Scale Short Form, Mini-Mental State and Confusion Assessment Method were used to identify patients with depression, dementia and delirium on admission, respectively. Barthel Index (BI) was used to evaluate patients’ functional status on admission, at discharge, 30-day, 90-day and 180-day after discharge. Generalized Estimating Equation (GEE) was used to calculate the associations between 3 D’s and BI.

**Results:**

One-hundred-and-forty-nine patients were included in this study. Twenty-seven patients (18.1%) had depression, 37 (24.8%) had dementia, and 85 (57.0%) had delirium. The study demonstrated that all the geriatric patients with functional decline presented gradual improvements of physical function up to 180 days after discharge. Whether depression exists did not substantially affect functional recovery after discharge, whilst either dementia or delirium could impede elder people functional status. The recovery of functional improvement in delirium or dementia was relatively irreversible when comparing with depression. Once delirium or dementia was diagnosed, poorer functional restore was expected. In brief, intensive work and strategies on modifying delirium or dementia should be put more effort as early as possible.

**Conclusions:**

Old hospitalized patients with depression can recover well after adequate intervention. We emphasize that early detection of dementia and delirium is imperative in subsequent functional outcome, even if at or before admission. Comprehensive plan must be implemented timely.

## Background

Functional decline, defined as deterioration in self-care skills, is a common and devastating problem for hospitalized elderly patients [1, 2]. It is associated with prolonged hospital stay, increased mortality, higher rates of institutionalization, and greater health care expenditure. Recent studies suggested that 34 to 50% of elderly patients experienced functional decline during hospitalization [3, 4]. The reasons for decline are often irrelevant to the patients’ admission diagnoses, but related to the underlying primary illness or iatrogenic complications during hospitalization [5, 6].

Geriatric syndromes are prevalent among older people and have been known to be associated with poor outcomes, such as readmission, increased length of stay, functional decline, hospitalization and mortality [7–10]. The most common geriatric syndromes include malnutrition, incontinence and geriatric psychiatric problems, in particular, depression, dementia and delirium [11]. The geriatric psychiatry--delirium, dementia, and depression, recognized as geriatric three D’s, frequently coexist and overlap by symptoms and caused clinical diagnosis challenging. Dementia is irreversible, and can only be prescribed by certain medication which is not beneficial in treatment outcome literally. The strategy for handling dementia should take environmental factors into consideration. Other than dementia, delirium and depression could be treated and reversible. Nevertheless, the presentation of hypoactive delirium or major depressive disorder were vague and can be easily confused by dementia. Three D’s symptoms often coexist. Once each of symptoms occurred in one old person simultaneously, the delay of diagnosis with proper intervention confer risk of functional recovery, which will lead to poor management of adverse outcomes. Hence geriatric psychiatric problems remain the most differential diagnoses in the older populations [12].

Understanding older patients who are at risk of functional decline during hospitalization is necessary before preventive strategies can be developed. Older age, lower Mini–Mental State Examination (MMSE) score, and poor nutrition status predict functional decline and deterioration of functional capacity restoration [2]. Geriatric conditions impede elder function after discharge had been well documented, several studies proposed certain useful predictors and provide unique tool in these field [8, 13–15]. Geriatric psychiatry had profound deterioration in functional outcome even several months after discharge, especially when co-occurrence of three D’s. Based on the past literature, we aim to discover whether functional restoration been affected by geriatric three D’s after hospitalization up to 180 days. In this study, we explore the impact of depression, dementia and delirium on functional trajectory in the hospitalized elder patients. To the best of our knowledge, this is the first study to elucidate the role of geriatric psychiatric three D’s on functional capacity 6 months after discharge.

## Materials and methods

### Study design and population

**Fig. 1:**
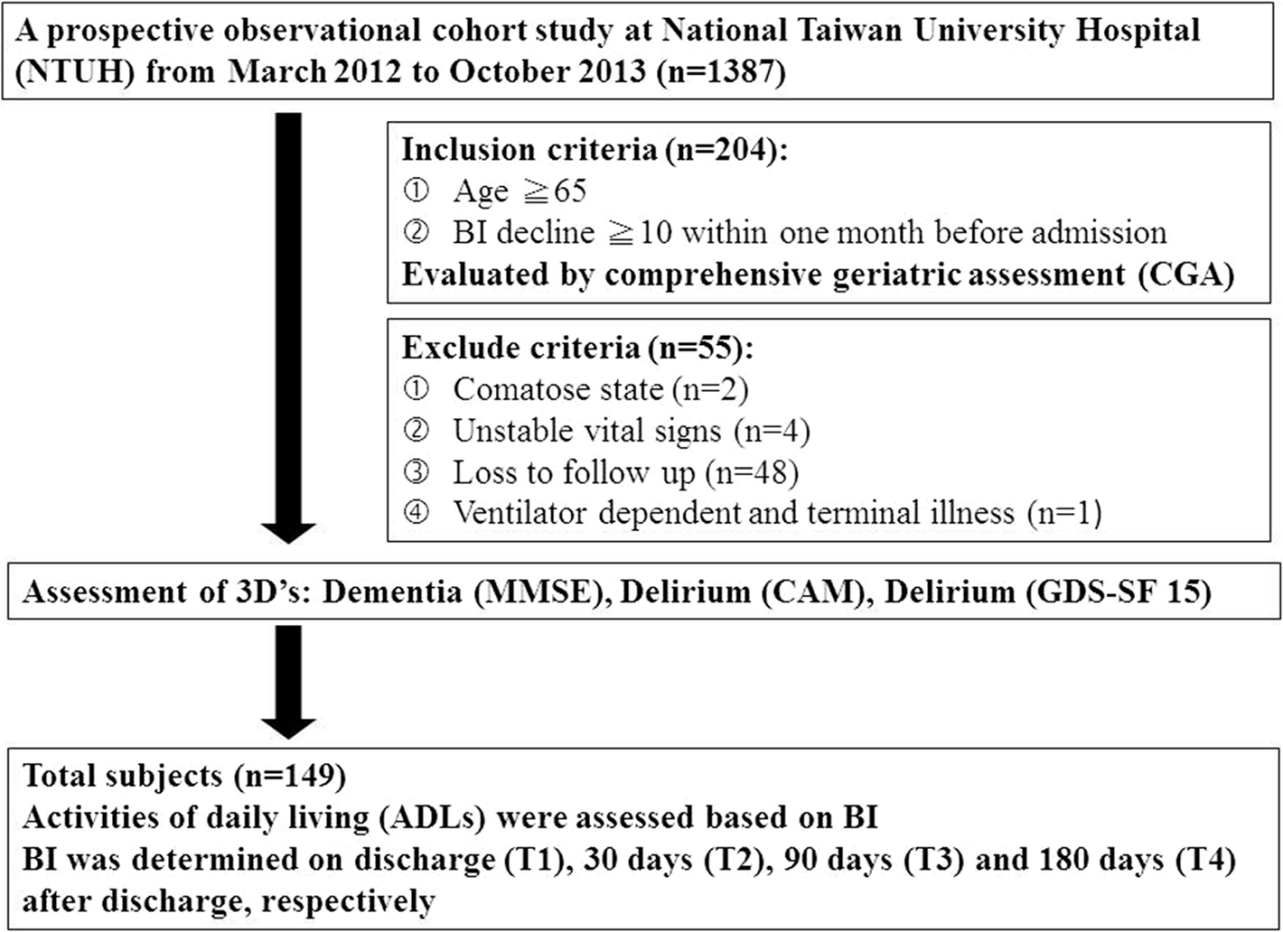
Flowchart of the subject enrollment. (ADLs = activities of daily living; BI: Barthel Index; CAM: Confusion Assessment Method; GDS-SF 15: Geriatric Depression Scale Short Form; MMSE: Mini–Mental State Examination)

A prospective observational cohort study was conducted at National Taiwan University Hospital (NTUH) from March 2012 to October 2013. A total of 149 hospitalized patients were invited to this study. All the participants were evaluated using comprehensive geriatric assessment (CGA) [16, 17] by the geriatric interdisciplinary team. Inclusion criteria were hospitalized patients aged 65 years or older, having a Barthel Index (BI) decline more than ten points within one month before the index hospitalization (the measurement of BI decline was recorded by questionnaire when admission). Exclusion criteria were patients who were in comatose state, unstable vital signs, ventilator dependent and terminal illness (e.g. multiple organ failure, cancer with or without metastatic lesion) (Fig. 1). The patients or their proxies were informed about the details of the present study and the potential risks during the process by the clinicians, and they were provided with the written informed consents. The proxies referred to the adult children, close relatives and friends of the patients. All the participants enrolled in the study signed the informed consent form by himself/ herself or their proxies. The research ethic committee at NTUH approved the study (No. 201108057RC).

### Data collection

#### Assessment of 3Ds

Depression was assessed according to Geriatric Depression Scale Short Form 15 (GDS-SF 15). Patients who scores more than 5 points are considered depressed [18]. Dementia or cognitive impairment was assessed according to Mini–Mental State Examination (MMSE), is a 30 points questionnaire to evaluate cognitive function with education adjusted cut-off points. Any score of 24 or more indicates normal cognition. Scores ≤9 points indicate severe, 10 to18 points was moderate, and 19 to 23 points was mild cognitive impairment [19]. Delirium was evaluated according to Confusion Assessment Method (CAM). The CAM diagnosis is based on four features: 1) acute onset and fluctuating course, 2) inattention, 3) disorganized thinking, and 4) altered level of consciousness. A diagnosis of delirium according to the CAM requires the presence of features 1, 2, and either 3 or 4 [20]..

#### Assessment of comorbidity and other geriatric problems

Patients who reported having comorbid illnesses such as hypertension, diabetes mellitus, stroke, coronary artery disease, chronic obstructive pulmonary disease, heart failure, atrial fibrillation, hip fracture, and Parkinson’s disease were recorded. Charlson Comorbidity Index (CCI) was also used to measure burden of disease [21].

Among other common geriatric problems, number of fall within one year prior to index admission was recorded. Hearing impairment is defined as having communication difficulties because of poor hearing with or without hearing aids. The visual impairment is conceptualized as poor eyesight despite using corrective lens.

#### Assessment of functional status

Activities of daily living (ADLs) (i.e. eating, transferring from bed to wheelchair, toileting, bathing, dressing, ambulation, and urination and defecation control) were assessed based on BI, and the total score of BI is 100 [22]. Ascertainment of functional decline is defined as BI decreases more than ten points in one month before admission. BI was also determined on discharge (T1), 30-day (T2), 90-day (T3) and 180-day (T4) after discharge (Fig. 1).

### Statistical analysis

Data were reported as number (percentage) for categorical variable and mean (standard deviation) for continuous variables. Group difference was assessed using chi-square or t-test when appropriate. The relationship between the development of ADL independence over time and the type of 3Ds was analyzed using generalized estimating equation (GEE).

The interaction terms of each geriatric condition by time points (using discharge as reference category) were added to the GEE model adjusted with age and gender. The changes in functional status are significantly different for a given subgroup (of a certain geriatric condition) when a significant interaction term appears.

Finally, to investigate the associated factors with functional status over time, GEE was adopted again in which all the patients’ characteristics were adjusted including depression, dementia and delirium into one model. All the statistical analyses were conducted using SPSS software version 15 (SPSS Inc., Chicago, Illinois). Statistical significance was defined as *p* < 0.05.

## Results

### Subjects

Table 1 depicts the clinical characteristics of all participants. The mean age was 87.6 years. Among the participants, 80.5% were 81 years or older, 54.4% were female, 60.4% received less than 9 years of education, 46.3% were still married, 10.7% living alone, and 69.8% having their sons or daughters as their primary contact person. The most common comorbidities were hypertension (78.5%), stroke (60.8%), diabetes mellitus (39.9%), coronary artery disease (26.4%), and chronic obstructive pulmonary disease (15.5%). On average, each subject had 4 comorbidities and the CCI revealed a score of 4.1.

**Table 1:** Characteristics of study sample (*N* = 149)

Characteristics	*N* (%)
Sociodemographic	
Age, years, Mean ± SD)	81.8 ± 7.8
< 80	29 (19.5)
81–90	64 (43.0)
> 90	56 (37.5)
Gender	
Male	62 (41.6)
Female	87 (54.4)
Education < 9 years	90 (60.4)
Marital status	
Married	68 (46.3)
Widowed	77 (51.7)
Others	3 (2.0)
Living	
Living alone	16 (10.7)
Living with family	131 (87.9)
Others	2 (4.7)
Primary contact person	
Self	24 (16.1)
Spouse	21 (14.1)
Children	104 (69.8)
Comorbidity	
Hypertension	117 (78.5)
Diabetes mellitus	59 (39.9)
Stroke	90 (60.8)
CAD	39 (26.4)
COPD	23 (15.5)
Heart failure	22 (14.9)
Atrial fibrillation	21 (14.2)
Malignancy	9 (6.2)
Hip fracture	20 (13.5)
Parkinson’s disease	29 (20.0)
No. of comorbidities (Mean ± SD)	4.08 ± 1.78
CCI score (Mean ± SD)	4.10 ± 2.47
Geriatric problems	
Fall incidence in the past year	81 (54.7)
Hearing impairment	17 (11.5)
Visual impairment	43 (29.1)
Geriatric condition	
No. of medications (Mean ± SD)	8.5 ± 4.1
Length of stay, days (Mean ± SD)	13.8 ± 10.2

*CAD* coronary artery disease, *CCI* Charlson comorbidity index, *COPD* chronic obstructive pulmonary disease, *LOS* length of stay

More than half of the subjects (54.7%) experienced fall in the past years, and 11.5 and 29.1% of the participants had hearing and visual impairments, respectively. On average, each participant took 8.5 prescription drugs per day and the length of hospitalization was 13.8 days (Table 1).

### Comparison of functional status among the 3D’s patients

**Fig. 2:**
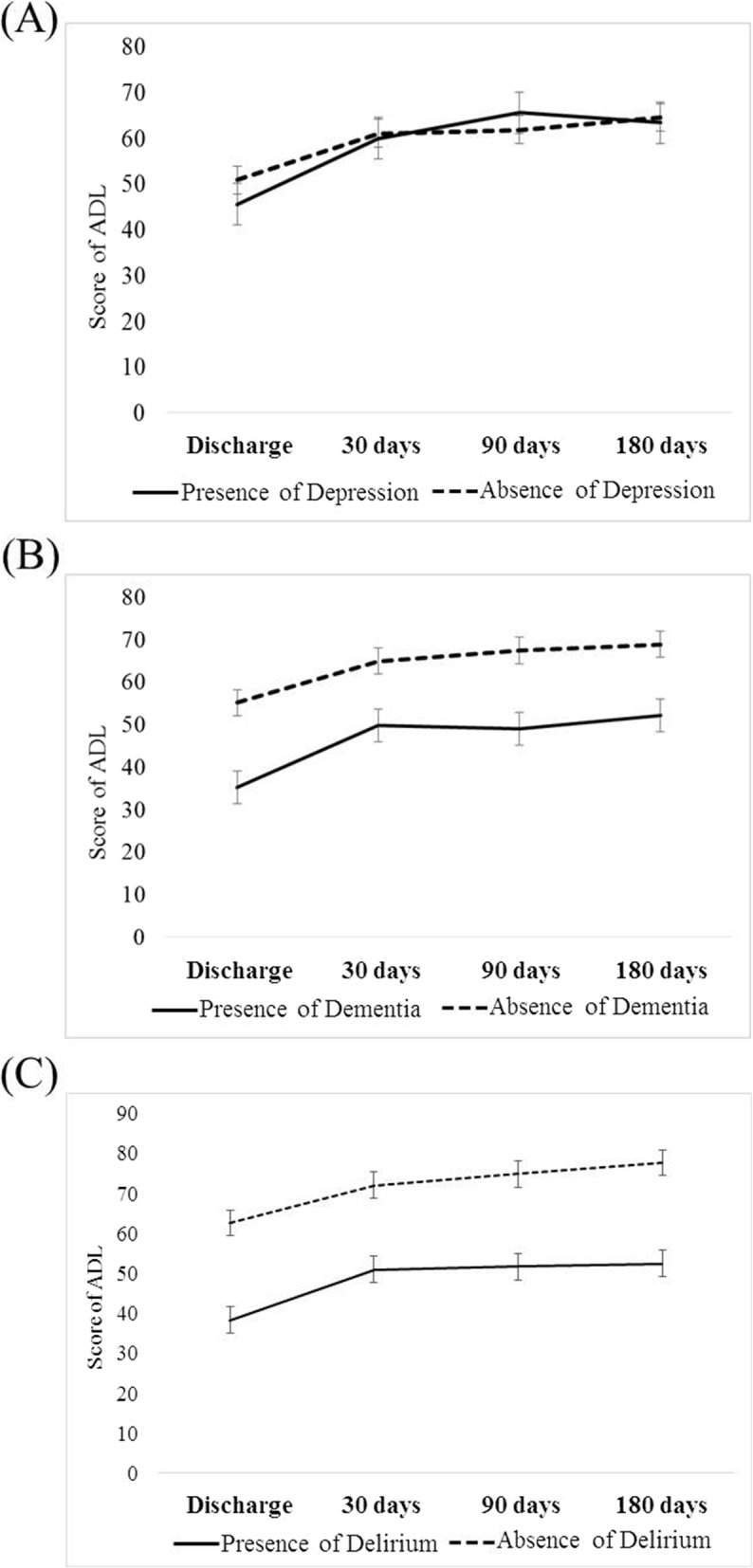
Mean score of ADL over time in the patients with or without (**a**) depression, (**b**) dementia, and (**c**) delirium

Of the 149 participants, 27 (18.1%) had depression, 37 (24.8%) had dementia, and 85 (57.0%) had delirium. The BI scores were similar among patients with or without depression in each time point even to the end of study time at 180 days. In contrast, patients with dementia or delirium demonstrated lower BI scores than those without and that the parallel gap persisted constantly up to six months after discharge (*p* < 0.01) (Table 2). This demonstrated a decreasing trend in functional regain after discharge (Table 2, Fig. 2 a-c). GEE was used to estimate the change in BI scores at various time points. Regardless of the presence of three D’s, the magnitude of functional improvement was not obvious in each time points in the three groups (Table 3, Fig. 2 a-c), When taking age, gender, dementia, delirium and depression into account in each model of three D’s, depression is independent from all the variables. Of note, in model of dementia group, delirium showed statistically significant (*p* = 0.003), and vice versa in model of delirium group (*p* = 0.004) (Table 3). In general, patients with delirium or dementia had worse functional status compared with those without, even though slightly improvement after discharge. On contrary, functional status remained similar to six months after discharge irrespective of depressive status.

**Table 2:** Comparison of ADL score in each time point according to difference emotional status (*N* = 149)

Variable	Maximum range	Actual range	T1 (*n* = 149)	T2 (*n* = 137)	T3 (*n* = 131)	T4 (*n* = 113)
Depression						
No	0–100	0–90	50.9 ± 28.8	61.1 ± 31.1	61.9 ± 30.6	64.6 ± 30.4
Yes	0–100	0–85	45.6 ± 26.0	60.0 ± 30.8	65.6 ± 30.3	63.4 ± 29.2
*P* value			*0.824*	*0.946*	*0.437*	*0.953*
Dementia						
No	0–100	0–70	55.2 ± 28.0	65.0 ± 31.1	67.5 ± 29.8	68.9 ± 29.5
Yes	0–100	0–90	35.2 ± 23.6	49.8 ± 27.9	49.1 ± 28.4	52.2 ± 28.5
*P* value			*0.001*	*0.003*	*0.001*	*0.001*
Delirium						
No	0–100	0–80	62.6 ± 27.2	72.0 ± 27.8	74.8 ± 27.0	77.6 ± 25.4
Yes	0–100	5–90	38.3 ± 24.1	50.9 ± 30.3	51.6 ± 29.3	52.4 ± 29.1
*P* value			*0.001*	*0.001*	*0.001*	*0.001*

T1: at discharge. T2: 30 days. T3: 90 days. T4: 180 days

**Table 3:** Multivariate GEE model for the Barthel Index score measures at discharge, and 30, 90, and 180 days follow-up (n = 149)

Variables	Model for Depression			Model for Dementia			Model for Delirium		
	Beta	SE	*P-value*	Beta	SE	*P-*value	Beta	SE	*P-*value
Constant	5.85	3.82	*0.125*	4.249	3.353	*0.205*	2.812	2.751	*0.307*
Time (T2-T1)	0.014	0.028	*0.622*	0.018	0.023	*0.444*	0.020	0.021	*0.346*
Time (T3-T1)	0.068	0.038	*0.075*	0.025	0.021	*0.234*	0.004	0.020	*0.840*
Time (T4-T1)	0.032	0.031	*0.303*	0.021	0.024	*0.337*	0.021	0.022	*0.342*
Age	0.043	0.045	*0.334*	0.019	0.040	*0.639*	0.030	0.033	*0370*
Gender	0.688	0.688	*0.303*	0.379	0.572	*0.508*	0.012	0.515	*0.981*
Depression	–	–	*–*	0.843	0.660	*0.201*	0.464	0.668	*0.487*
Dementia	1.020	0.704	*0.148*	–	–	*–*	1.813	0.626	*0.004*
Delirium	0.441	0.69	*0.523*	1.839	0.628	*0.003*	–	–	*–*

Age and Gender have been controlled in all models

All models had random intercepts

All time-dependent covariates had a fixed slope

T1: at discharge; T2: 30 days; T3: 90 days; T4: 180 days

Beta: non-standardized regression coefficient in multilevel analyses; SE: standard error

## Discussion

The present study demonstrated that all the geriatric patients with functional decline presented gradual improvements of physical function up to 180 days after discharge. Whether depression exists did not substantially affect functional recovery after discharge, whilst either dementia or delirium could impede elder people functional status.

The term “geriatric syndromes” contains the features of various conditions in the elder people. The causes of geriatric syndromes is multifactorial, patient-specific and situation-specific, and often lead to subsequent sequela, morbidity and poor outcomes in hospitalized elderly [10, 23]. Geriatric psychiatric problems—delirium, dementia, and depression denote the most common presentation and obscure diagnoses for older adults [12]. These syndromes may overlap and exist simultaneously or emerge exclusively in one patient, and affect or confer to the other mutually, which eventually lead to functional decline, institutionalization and even death [12, 24].

Old people suffering from one of the geriatric psychiatric problems often have poor ADL outcomes and increasing risks of death. Several studies have developed strategies to predict the outcomes of functional decline or mortality in the elderly [13, 14]. Barnes et al proposed a new strategy for prognosis, which can predict the risk of outcomes including functional recovery, dependence and death [15]. McCusker et al investigated the co-occurrence of the delirium, depression and dementia, and found that those without co-occurrence had better outcome [25]. In our study, we stratified functional decline of the hospitalized elder patients on admission and assessed the association between geriatric psychiatric problems and functional change after discharge. The patients with delirium were too confused to maintain self-care, thus a low BI score were expected. Dementia is a strong risk factor for delirium, and once delirium develops, it can accelerate and worsen physical functions [26, 27]. Similar to other studies, our finding also showed that the presence of delirium and dementia was associated with poorer functional recovery after hospitalization [28–30]. Slightly parallel improvements in functional trajectories after discharge among those with or without delirium or dementia were seen (Fig. 2 a-c). Once dementia and delirium present before admission, each of them impedes elder people function persistently.

To overcome these functional gaps, patients may need to consider other interventions such as rehabilitations, or as in this study, CGA. CGA is a useful tool that involves interdisciplinary diagnostic process to identify functional, medical, mental, and socio-environmental complex problems of frail elderly in order to coordinate a proper program to treat and manage for optimal outcome [16, 17, 23]. CGA is specialized in geriatric wards than general ward with geriatric teams [16]. Furthermore, direct communication, highly trained staff and effective interdisciplinary team will implicate the treatment outcome in subspecialized geriatric ward and inpatient stroke care unit [31]. CGA during hospitalization has effect on decreasing mortality, improvement function, and decreasing placement in nursing homes. The prolong effect after acute illness also reveal good impact, especially in at-risk elderly in the community [32]. Our results disclose that patients’ functional status improved regardless of their geriatric psychiatric problems, suggesting the effectiveness of CGA.

On the other hand, all depressed patients improved functional outcome and as equivalent to those without depression up to 6 months after discharge, suggesting depression is a reversible disease and early intervention is crucial in reversing the functional outcome. Previous researches have highlighted the under diagnosis of depression among the elderly [33]. Although diagnosis is challenging, it is nevertheless potentially treatable morbidity in older people [34–36], clinical practitioner should put efforts on access to appropriate treatment. In our study, the magnitude of ADL improvement is equal in depression group after 180 days discharge. The explanations are first, the awareness of depression by caregiver that helps in coping with depression either in daily activity or taking antidepressant agents. Second, delirium and dementia have greater impact impeding functional status, whilst depression mainly influences mental function but not physical function.

Functional outcome in elder people are thought to be affected especially with more comorbidities, older age or more geriatric syndromes. Whether patients recover or maintain ADLs at an optimal state depends largely on careful assessments and plans. It is imperative that elderly patients with geriatric psychiatric condition, especially delirium and dementia should undergo rehabilitation in order to improve physical functioning. We highlighted CGA during admission in elder people, and the application was suggested effective and crucial during the whole course after discharge. The recovery of functional improvement in delirium or dementia was relatively irreversible when comparing with depression. Given the diagnosis of delirium or dementia was established, poorer functional restore was utterly expected. Hence, intensive work and strategies on modifying delirium or dementia should be put more effort as early as possible, not only by medical stuff but primary caregiver. Readily intervention with accuracy diagnosis of dementia and delirium is important. We suggest further research focusing on which intervening on depression in post-hospitalization care regain promising better functional outcomes. And, if so, whether pharmacologic or non-pharmacologic plan could lead to functional recovery should be investigated.

This study has several limitations. First, the sample size was relatively small, thus the confounding variables could not draw a distinct effect on each of the 3D’s in terms of functional outcomes. Second, the patients were not evaluated by the same staff every time during CGA, thus differences in reporting data and inconsistency may occur. Reassessed using rigorous research methods with well-trained same staff can provide much more promising results. Third, the definition of each of the 3D’s might also be underestimated of the true prevalence in this study. Forth, the population had a substantial loss of follow-up for certain reasons such as rehospitalization may diminish the statistic power in this study.

## Conclusion

In conclusion, old hospitalized patients with depression can recover well after adequate intervention. We emphasize that early detection of dementia and delirium is imperative in subsequent functional outcome, even if at or before admission. Comprehensive plan must be implemented timely.

## Data Availability

All data generated or analyzed during this study are included in this published article.

## Abbreviations

3D’s: Three geriatric conditions, including depression, dementia and delirium
ADLs: Activities of daily living
BI: Barthel Index
CGA: Comprehensive geriatric assessment
